# Oral Administration of *Lactobacillus plantarum* Strain AYA Enhances IgA Secretion and Provides Survival Protection against Influenza Virus Infection in Mice

**DOI:** 10.1371/journal.pone.0086416

**Published:** 2014-01-22

**Authors:** Yosuke Kikuchi, Ayami Kunitoh-Asari, Katsuyuki Hayakawa, Shinjiro Imai, Kenji Kasuya, Kimio Abe, Yu Adachi, Shin-ichi Fukudome, Yoshimasa Takahashi, Satoshi Hachimura

**Affiliations:** 1 Research Center for Basic Science, Research and Development, Quality Assurance Division, Nisshin Seifun Group Inc., Fujimino-city, Saitama, Japan; 2 Graduate School of Nutritional and Environmental Sciences, University of Shizuoka, Shizuoka-city, Shizuoka, Japan; 3 Yeast Function Development Unit, Oriental Yeast Co., Ltd., Itabashi-ku, Tokyo Japan; 4 Laboratory of Yeast and Fermentation Food Division, Oriental Yeast Co., Ltd., Itabashi-ku, Tokyo, Japan; 5 Department of Immunology, National Institute of Infectious Diseases, Shinjuku-ku, Tokyo, Japan; 6 Research Center for Food Safety, Graduate School of Agricultural and Life Sciences, University of Tokyo, Bunkyo-ku, Tokyo, Japan; Virginia Tech University, United States of America

## Abstract

The mucosal immune system provides the first line of defense against inhaled and ingested pathogenic microbacteria and viruses. This defense system, to a large extent, is mediated by the actions of secretory IgA. In this study, we screened 140 strains of lactic acid bacteria for induction of IgA production by murine Peyer’s patch cells. We selected one strain and named it *Lactobacillus plantarum* AYA. We found that *L. plantarum* AYA-induced production of IL-6 in Peyer’s patch dendritic cells, with this production promoting IgA^+^ B cells to differentiate into IgA-secreting plasma cells. We also observed that oral administration of *L. plantarum* AYA in mice caused an increase in IgA production in the small intestine and lung. This production of IgA correlated strongly with protective ability, with the treated mice surviving longer than the control mice after lethal influenza virus infection. Our data therefore reveals a novel immunoregulatory role of the *L. plantarum* AYA strain which enhances mucosal IgA production and provides protection against respiratory influenza virus infection.

## Introduction

Influenza virus (IFV) infection is a significant cause of morbidity and mortality worldwide. The constant threat of the emergence of a novel influenza subtype engenders an even greater risk to society. Therefore, it is important to enhance local immunity to decrease the risk of IFV infection [1]. The mucosal immune system provides the first line of defense against inhaled and ingested pathogenic microbacteria and viruses. This defense system, to a large extent, is mediated by the actions of secretory IgA [2], which is the most abundantly produced Ig isotype in the body [3]. The primary role of mucosal IgA is to neutralize inhaled bacteria and viruses by interfering with their motility or by inhibiting their adherence to epithelial cells [4]. Secretary IgA antibodies in the mucosa are therefore believed to provide primary defense against respiratory IFV infection [5], although studies using IgA (−/−) mice have shown that other compensatory mechanisms may also be involved in this protection [6].

Recognition of IFVs through pattern recognition receptors plays a central role in the generation of adaptive immune responses. Recently, Ichinohe et al. [7] reported that commensal bacteria, which maintain immune homeostasis in the intestine, regulate immunity in the respiratory mucosa through proper activation of inflammasomes. Their data demonstrated that some commensal bacteria also contribute to immunocompetence in the lung. Oral and intranasal administration of lactic acid bacteria (LAB) has been shown to protect against IFV [8], [9]. However, the mechanism by which LAB enhances protection against IFV infection remains unclear. Using information from previous reports, the present study attempted to provide protection against IFV by oral administration of LAB and focused on the effect of LAB on IgA production. We first screened LAB strains to obtain a strain with the highest IgA-inducing activity in Peyer’s patches (PPs) and then determined the mechanism by which this LAB-induced IgA production.

IgA-secreting mucosal plasma cells originate mainly from homing IgA-committed B cells, which undergo IgM-to-IgA isotype class switching at inductive sites of mucosal immunity, such as PP and nasopharynx-associated lymphoid tissue (NALT) [10]–[12]. This process of B-cell differentiation, including class switching, is essential for inducing IgA expression at the mucosal surface. Litinskiy et al. [13] showed that dendritic cells (DCs) upregulated B-cell–activating factor (BAFF) and a proliferation-inducing ligand (APRIL) leading to class switching to IgA. It is well established that mucosal DCs enhance IgA production through factors such as IL-6, retinoic acid, and NO [14]–[16]. This led us to hypothesize that DCs may play a key role in the promotion of IgA production by LAB.

In this study, we examined the role of DCs in the IgA-enhancing effect of a LAB strain and also investigated whether oral administration of LAB activated the immune system of the lung and protected against IFV infection.

## Materials and Methods

### Mice

Female BALB/c mice aged 6–10 weeks and weighing 18–25 g were obtained from Japan SLC Inc. (Shizuoka, Japan). All the mice were housed under specific pathogen-free conditions. Ten mice were housed in each plastic polypropylene cage. They were provided an experimental diet and water *ad libitum* under a 12-h light–dark cycle. The mice were divided into two experimental groups with similar mean body weight. All the animal studies described in this paper were approved by the Animal Care and Use Committee of the National Institute of Infectious Diseases (approval ID; 110006) or Nisshin Seifun Group Inc. Ltd (approval ID; GA1002, GA1003, GA1005).

### Bacterial Strain and Culture Conditions

The LAB were obtained from the culture collection of Oriental Yeast Co., Ltd (Table 1) and cultured in sterile GYP broth (1% glucose, 1% yeast extract, 0.5% Bacto-peptone, 0.2% sodium acetate⋅3H_2_O, 20 ppm MgSO_4_⋅7H_2_O, 1 ppm MnSO_4_, 1 ppm FeSO_4_⋅7H2O, 1 ppm NaCl, 2.5 ppm Tween 80, pH6.8). The cells were harvested by centrifugation at 5000×*g* for 10 min and then washed three times with sterile saline solution. The washed cells were sterilized in an autoclave and then lyophilized. Therefore, all LAB strain samples used in this study are killed bacteria preparations.

**Table 1:** Species of LAB.

number	strain
NO. 1–11	*Lactobacillus brevis*
NO. 12–21	*Pediococcus pentosaceus*
NO. 22–27	*Lactobacillus pentosus*
NO. 28–74	*Lactobacillus plantarum*
NO. 75	*Lactobacillus buchneri*
NO. 76–77	*Lactobacillus casei subsp.casei*
NO. 78–79	*Lactobacillus casei subup pseudplantarum*
NO. 80	*Lactobacillus coryniformis subsp. Coryniformis*
NO. 81–87	*Lactobacillus curvatus*
NO. 88–89	*Lactobacillus fermentum*
NO. 90	*Lactobacillus fructosus*
NO. 91–92	*Lactobacillus gasseri*
NO. 93	*Lactobacillus helveticus*
NO. 94–95	*Lactobacillus hilgardi*
NO. 96	*Lactobacillus kefirgranum*
NO. 97	*Lactobacillus kfiri*
NO. 98–99	*Lactobacillus mali*
NO. 100–101	*Lactobacillus murinus*
NO. 102	*Lactobacillus para paracasi*
NO. 103	*Lactobacillus parabuchineri*
NO. 104	*Lactobacillus paracasei subsp. Tolerans*
NO. 105–106	*Lactobacillus paracasei subsp.paracasei*
NO. 107	*Lactobacillus parakefiri*
NO. 108	*Lactobacillus viridesceus*
NO. 109–111	*Leuconostoc citreum*
NO. 112	*Leuconostoc lactis*
NO. 113–115	*Leuconostoc mes*
NO. 116–123	*Leuconostoc mesenteroides*
NO. 124	*Leuconostoc mesenteroides subsp.cremoris*
NO. 125–127	*Leuconostoc mesenteroides subsp.dextranicum*
NO. 128–129	*Leuconostoc mesenteroides subsp.mesenteroides*
NO. 130–133	*Leuconostoc paramesenteroides*
NO. 134	*Leuconostoc carnosum*
NO. 135	*Leuconostoc fallax*
NO. 136	*Leuconostoc psudemesenteroides*
NO. 137	*Pediococcus acidilactici*
NO. 138	*Pediococcus damnosus*
NO. 139	*Pediococcus acidilactici*
NO. 140	*Weis halotolarans*

### Isolation of PP Cells

BALB/c mice were sacrificed by cervical dislocation after a one-week acclimatization period. PP cells were collected from the side of the small intestine and incubated in RPMI 1640 (Sigma-Aldrich, St. Louis, MO, USA) with 10% FCS (Sigma-Aldrich) and 0.2 units of collagenase type I (1 mg/ml; Sigma-Aldrich) on a magnetic stirrer at 37°C for 60 min. The cell suspensions were then passed through a 70-µm nylon cell strainer (BD Biosciences, San Jose, CA, USA), centrifuged at 4°C for 5 min, and washed with PBS. Then, they were vigorously shaken for 10 s and centrifuged at 4°C for 5 min. After two further washes, the cells were cultured in RPMI 1640 supplemented with 10% FCS, 1% glutamine, 1% penicillin, and 1% streptomycin. This procedure yielded a population of PP cells.

### Flow Cytometric Analysis

PP cells (1×10^6^) in 100 µl of FACS buffer (PBS containing 0.5% BSA and 2 mM EDTA) were incubated with 10 µl of CD16/32 (Miltenyi Biotec, Auburn, CA, USA), mixed well, and refrigerated for 10 min. The cells were then washed with 1 ml of FACS buffer and centrifuged at 300×*g* for 10 min. The supernatant was aspirated completely and a further 100 µl of FACS buffer added to the cells. The cell suspension was incubated with 10 µl of anti-IgA–FITC antibody (Miltenyi Biotec), mixed well, and incubated for a further 10 min in the dark in a refrigerator (2°C–8°C). The cells were then washed with 1 ml of FACS buffer and centrifuged at 300×*g* for 5 min. The supernatant was aspirated completely and the cells were resuspended in 100 µl of FACS buffer, followed by incubation with 10 µl of PE rat anti-mouse CD45R/B220 (BD Pharmingen, San Diego, CA, USA). After thorough mixing, the cells were incubated for 10 min in the dark in a refrigerator (2°C–8°C), washed again with 1 ml of FACS buffer, and then centrifuged at 300×*g* for 5 min. The supernatant was aspirated completely and the cells were resuspended in FACS buffer. Analysis of the cells was carried out using a FACSCanto (BD Biosciences), with the data obtained by FlowJo ver. 8 (Tree Star, Ashland, OR, USA).

### Collection of Small Intestinal Washings

BALB/c mice were sacrificed by cervical dislocation and the small intestine was collected. The small intestine was washed by flushing with PBS containing protease inhibitors (Roche Diagnostics, Rotkreuz, Switzerland). The fluid was collected and centrifuged at 9200×*g* for 30 min at 4°C, then the supernatant was collected as the small intestinal washings.

### Separation of IgA^+^ B Cells from PP Cells

PP cells (1×10^7^) in 100 µl of magnetic-activated cell sorting (MACS) buffer (PBS containing 5% BSA and 2 mM EDTA) were incubated with 10 µl of anti-IgA–FITC Ab (Miltenyi Biotec), mixed well, and incubated for 20 min in the dark in the refrigerator (2°C–8°C). The cells were then washed with 1 ml of MACS buffer and centrifuged at 300×*g* for 5 min. The supernatant was aspirated completely and 100 µl of MACS buffer added to the cells. The cell suspension was incubated with 10 µl of anti-FITC microbeads (Miltenyi Biotec), mixed well, and incubated for a further 20 min in the dark in the refrigerator (2°C–8°C). The cells were washed again with 1 ml of MACS buffer and centrifuged at 300×*g* for 5 min. The supernatant was aspirated completely, and the cells were resuspended in 100 µl of MACS buffer. An LS column adapter was inserted into the magnetic field of the VarioMACS separator (Miltenyi Biotec), and the cell suspension was applied to the LS column (Miltenyi Biotec). The column was then washed three times with 3 ml of MACS buffer and the total effluent was collected for later separation of DCs from PP cells. The LS column was removed from the magnetic field, flushed with 1 ml of MACS buffer to remove the fraction of magnetically-labeled cells. These cells were regarded as IgA^+^ B cells separated from PP cells.

### Separation of DCs from PP Cells

A suspension of PP cells lacking IgA-positive cells (1×10^7^ cells) obtained from the above experiment that separated IgA^+^ B cells from PP cells (i.e., effluent from the LS column) was added to 50 µl of MACS buffer, followed by incubation with 2.5 µl of anti-CD11c microbeads (Miltenyi Biotec). The cells were mixed well, incubated for 20 min in the dark in the refrigerator (2°C–8°C), washed with 1 ml of MACS buffer, and then centrifuged at 300×*g* for 5 min. The supernatant was aspirated completely and the cells were resuspended in 50 µl of MACS buffer. An LD column adapter was inserted into the magnetic field of the VarioMACS separator and the cell suspension was applied to the column. The column was washed twice with 1 ml of MACS buffer and then removed from the magnetic field. MACS buffer (1 ml) was pipetted into the column to flush out the cell suspension. The LS column adapter was then inserted, followed by application of the cell suspension to the column. After three washings with 3 ml of MACS buffer, the column was removed from the magnetic field and 1 ml of CS buffer was added to flush out the cell suspension. These cells were regarded as DCs separated from PP cells.

### Cell Culture

The cells were cultured in a humidified 5% CO_2_ atmosphere in RPMI 1640 (Sigma-Aldrich) containing 10% FCS, 2 mM of L-glutamine, 100 units/ml of penicillin, and 100 µg/ml of streptomycin, referred to as complete medium. PP cells were plated at a density of 1×10^6^ cells/well, DCs separated from PP cells at a density of 1×10^4^ cells/well, IgA^+^ B cells separated from PP cells at a density of 1×10^5^ cells/well, and the LAB powder at a density of 50 µg/well.

### Administration of LABs

LABs were added to an AIN93G diet (Oriental Yeast Co., Ltd.). The control diet was the AIN93G diet without LABs. Mice were provided the diet and water *ad libitum*. Average consumption of the diet was 2.4 g/day. This resulted in an intake of 120 mg LAB/day.

### Real-time PCR Analysis of DC RNA

After incubation, the DCs were harvested and total RNA isolated using the RNAeasy Mini Kit (Qiagen, Hilden, Germany) according to the manufacturer’s instructions. cDNA was then synthesized from 250 ng of total RNA using the SuperScript VILO cDNA Synthesis Kit (Invitrogen, Carlsbad, CA, USA) according to the manufacturer’s instructions. cDNA was reverse transcribed from 500 ng total RNA. Real-time PCR was performed as follows. The reaction mixtures of 10 µl included 5 µl of SYBR Premix Ex Taq II (Takara, Shiga, Japan), 1 µM of target gene primer pairs, 1 µl of cDNA, and sterilized distilled water. The primers were purchased from Operon Biotechnologies (Tokyo, Japan). Real-time PCR was run at 95°C for 15 min and then for 45 cycles at 95°C for 15 s, 57°C for 30 s, 72°C for 15 s, and 72°C for 15 min. This reaction was carried out using a LightCycler (Roche Diagnostics). To standardize the target gene level, we used transcripts of glyceraldehyde 3-phosphate dehydrogenase (GAPDH), a housekeeping gene, as the internal control.

The sequences of the primers were as follows: IL-6, 5′-TGGAGTCACAGAAGGAGTGGCTAAG-3′ and 5′-TCTGACCACAGTGAGGAATGTCAAC-3′; TGF-β, 5′-ATTGAGGGCTTGTTGAGATG-3′ and 5′-GACTGGCGAGCCTTAGTTTG-3′; BAFF, 5′-TGCTATGGGTCATGTCATCCA-3′ and 5′-GGCAGTGTTTTGGGCATATTC-3′; GAPDH, 5′-TGAACGGGAAGCTCACTGG-3′ and 5′-TCCACCACCCTGTTGGTGTA-3′; inducible nitric oxide synthase (iNOS), 5′-CGTTGGATTTGGAGCAGAAGTG-3′ and 5′-CATGCAAAATCTCTCCACTGCC-3′; APRIL, 5′-TCACAATGGGTCAGGTGGTATC-3′ and 5′-TGTAAATGAAAGACACCTGCACTGT-3′; aldehyde dehydrogenase (ALDH)1a1, 5′-ATGGTTTAGCAGCAGGACTCTTC-3′ and 5′-CCAGACATCTTGAATCCACCGAA-3′; and ALDH1a2, 5′-GACTTGTAGCAGCTGTCTTCACT-3′ and 5′-TCACCCATTTCTCTCCCATTTCC-3′.

### Measurement of Cytokine and Ig Production

Cytokine and Ig production in the cell culture supernatants was assayed by specific sandwich ELISA using the following kits: mouse IgA ELISA quantitation set (Bethyl Laboratories, Montgomery, TX, USA), Quantikine Mouse/Rat/Porcine/Canine TGF-β Immunoassay (R&D Systems, Minneapolis, MN, USA), Quantikine Mouse BAFF/BLyS/TNFSF13B Immunoassay (R&D Systems), mouse IL-6 ELISA kit (Thermo Fisher Scientific, Waltham, Massachusetts, USA), mouse IgG1 ELISA quantitation set (Bethyl Laboratories), and mouse IgG2a ELISA quantitation set (Bethyl Laboratories). The kits were used according to the manufacturer’s instructions. Briefly, 96-well immunoplates were coated with antibodies. After washing and blocking the plates, the samples and standards were added, followed by incubation. After washing, horseradish peroxidase-labeled mAbs were added and the plates incubated. The plates were then washed again, incubated with the substrate, 3,3′,5,5′-tetramethylbenzidine, followed by addition of a stop solution and measurement of absorbance at 450 nm.

### Influenza Virus Infection

The X-31 (H3N2) virus was grown in the allantoic sacs of 10-day-old chicken embryos at 34°C for 3 days. The X-31 virus was purified from the allantoic fluid, and the virus suspension was stored at −80°C. Fifteen mice were anesthetized per group and infected by dropping 10 µl of suspension containing 5 LD_50_ of virus into each nostril (20 µl/mouse). After the virus challenge, the mice were housed under Biosafety Level 2 in the animal facility. We monitored body weight daily and set up the humane endpoint as 20% body weight loss after IFV infection. In some experiments, bronchoalveolar lavage fluid (BALF) was obtained 3 days after infection.

### Measurement of X-31 Specific IgA Production

The inactivated X-31 virus was suspended in PBS. Instead of a primary antibody in mouse IgA ELISA quantitation set (Bethyl), this purified virus was coated at 5 µg/ml on the ELISA plate. After this procedure, this kit was used according to the manufacturer’s instructions. IgA monoclonal antibody specific for X-31 virus was used as a standard. The detection limit was 31.25 ng/ml.

### Statistical Analysis

Student’s t test and Mann–Whitney’s U test were used to identify statistically significant differences between the test and control groups. The differences in survival rates were tested by the Kaplan–Meier test. P values of less than 0.05 were considered statistically significant.

## Results

### Screening of LAB Strains

**Figure 1:**
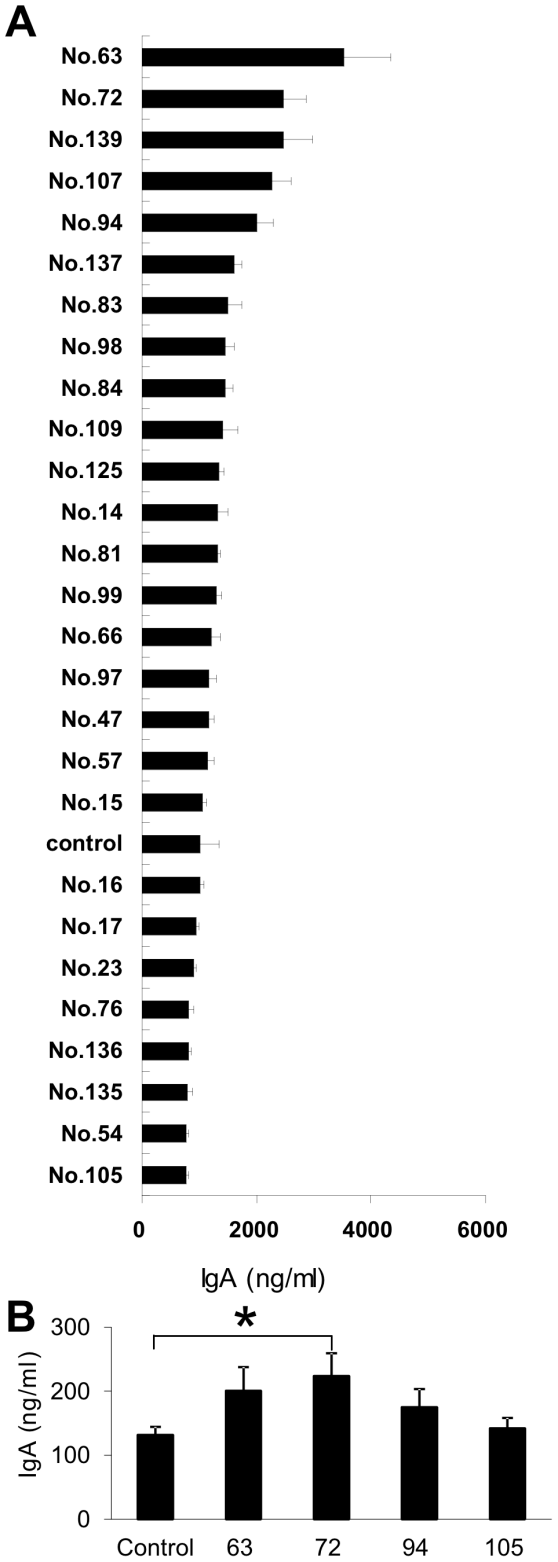
IgA production induced by PP cells. (A) PP cells (1×10^6^/well) from BALB/c mice fed a normal diet (AIN93G), and each LABs were co-cultured. Supernatants were harvested after 7 days, and IgA concentrations were measured by ELISA. Data are shown as mean ± SE. n = 5. Data represent one of two similar experiments. (B) PP cells (1×10^6^ per well) from BALB/c mice fed the diet containing LAB were incubated. Supernatants were harvested, and IgA concentrations were measured by ELISA after 7 days. Data are shown as mean ± SE. n = 5. Asterisk indicate unpaired Student’s t test; P value is <0.05. Data represent one of two similar experiments.

To obtain LAB strains with high infection defense activity, we screened 140 strains (Table 1) of this bacterial species for their ability to induce production of IgA in murine PP cells. First, we divided the 140 strains into five groups and compared induction of IgA production within each group. Five strains with the highest inducability were selected in each group. Twenty-seven strains were selected, two of which added as negative controls (data not shown). We compared the ability of these 27 strains to induce IgA production (Fig. 1A) and selected three strains (2 strains of *L. plantarum,* strain no. 63 and 72; and a strain of *L. hilgardi* (Strain no. 94). We selected the *L. paracasei* strain as the negative control (Strain no.105). *In vivo* production of IgA was investigated in these four strains. Strains 107 and 139 were not selected because both proved difficult to grow (data not shown).

*In vivo* screening of the four strains showed that mice fed strain 72 had the highest total IgA concentration in PP cell culture supernatants, and that only strain 72 showed statistical differences from the control (Fig. 1B). We therefore selected strain 72 and named it *L. plantarum* strain AYA (FERM P-21106).

### Ingestion of L. plantarum AYA Enhanced the Proportion of IgA^+^ B220^+^ Cells Present among PP Cells and IgA Production in the Small Intestine

**Figure 2:**
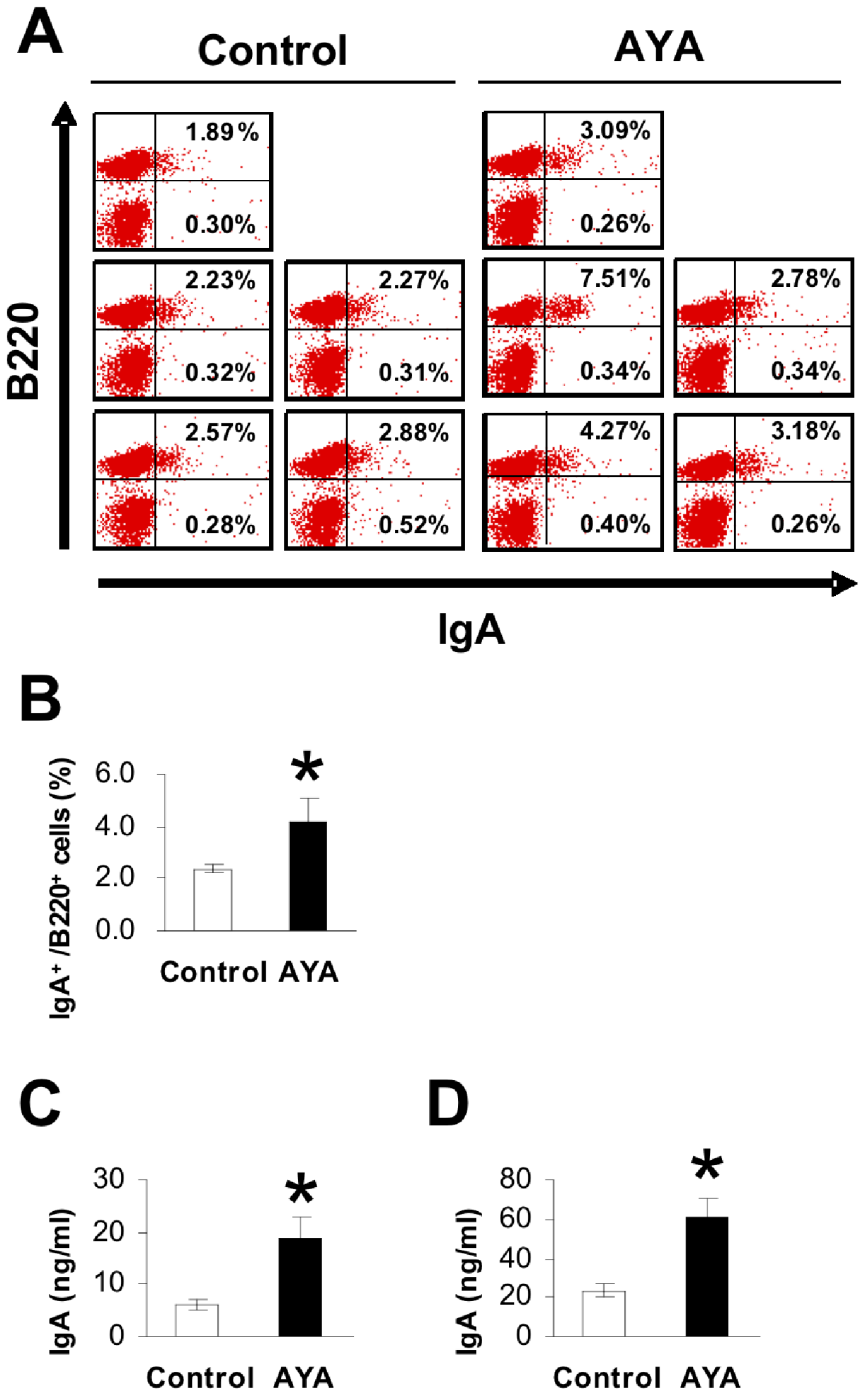
The proportion of IgA^+^B220^+^ cells present among PP cells was measured. IgA^+^ B220^+^ cells were from BALB/c mice fed the normal diet with or without LAB for 28 days. We used flow cytometry to measure surface IgA and B220 expression. (A) FACS profiles of PP cells. The position of IgA^+^ B220^+^ cells in Fig. 2A is located in the upper right zone. Data represent one of two similar experiments. (B) The proportion of IgA^+^ B220^+^ cells present among PP cells. Data are shown as mean ± SE. n = 5. Asterisk indicate unpaired Mann–Whitney’s U test; P value is <0.05. Data represent one of two similar experiments. (C) IgA concentration in the washings from the upper small intestine. Data are shown as mean ± SE. n = 5. Asterisk indicate unpaired Mann–Whitney’s U test; P value is <0.05. Data represent one of two similar experiments. (D) IgA concentration in the washings from the lower small intestine. Data are shown as mean ± SE. n = 5. Asterisk indicate unpaired Mann–Whitney’s U test; P value is <0.05. Data represent one of two similar experiments.

To study the cause of the increase in IgA production in PP cells following ingestion of *L. plantarum* AYA, we measured the ratio of IgA^+^ B220^+^ cells present among the PP cells. Fig. 2A–B shows the ratio of IgA^+^ B220^+^ cells to PP cells in BALB/c mice fed heat-treated *L. plantarum* AYA. The proportion of IgA^+^ B220^+^ cells was approximately two fold higher in mice fed *L. plantarum* AYA compared with those not inoculated with LAB. As shown in Fig. 2C–D, the IgA concentration in the small intestine of the *L. plantarum* AYA diet group was significantly higher than that of the control group (washings from the upper and lower small intestine, P<0.05 ).

### L. plantarum AYA-stimulated DCs can Directly Impact IgA^+^ B Cells to Promote IgA Production

**Figure 3:**
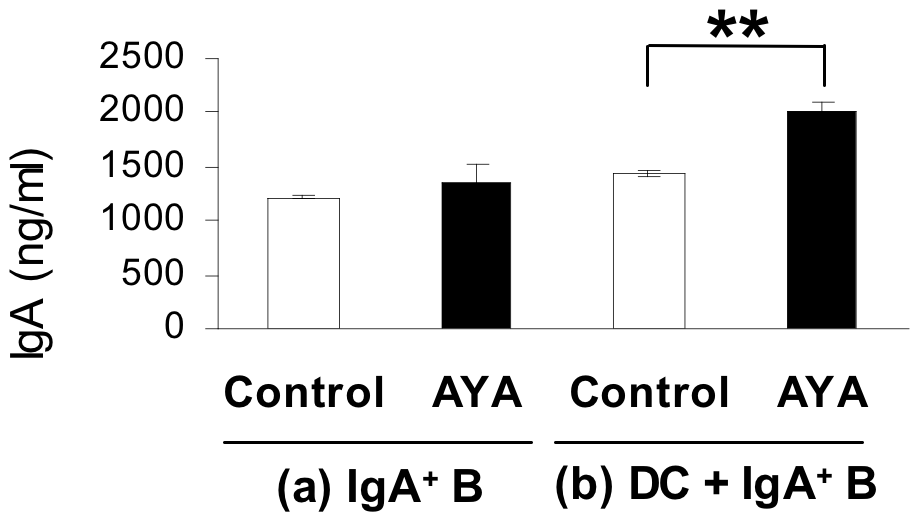
IgA production induced by IgA^+^ B cell with or without *L. plantarum* AYA and DC. (a) IgA^+^ B cells from PP cells were cultured in the absence or presence of *L. plantarum* AYA. IgA concentrations in the supernatants were determined by ELISA after 4 days. Data are shown as mean ± SE. n = 3. There were no statistically significant differences as determined by Student’s t test. Data represent one of two similar experiments. (b) DCs and IgA^+^ B cells from PP cells were cultured in the absence of presence of *L. plantarum* AYA. IgA concentrations in the supernatants were determined by ELISA after four days. Data are shown as mean ± SE. n = 3. Asterisk indicate unpaired Student’s t test; P value is <0.01. Data represent one of two similar experiments.

To determine the mechanism responsible for the increase in IgA production in PP cells following ingestion of *L. plantarum* AYA, we conducted the following tests. First, to investigate the direct impact of DCs on IgA antibody-enhancing effects of *L. plantarum* AYA, we established *in vitro* coculture assays. One group included IgA^+^ B cells separated from PP cells cultured in the presence or absence of *L. plantarum* AYA(Fig. 3a), and the other group included DCs and IgA^+^ B cells cultured in the presence or absence of *L. plantarum* AYA (Fig. 3b). The total IgA concentration in the culture supernatants of IgA^+^ B cells with *L. plantarum* AYA was not statistically different from that in supernatants of IgA^+^ B cells without *L. plantarum* AYA (Fig. 3a). On the other hand, total IgA concentration in culture supernatants of DCs and IgA^+^ B cells with *L. plantarum* AYA was higher than that in culture supernatants of DCs and IgA^+^ B cells without *L. plantarum* AYA (P<0.01) (Fig. 3b). These data indicate that DCs play an important role in the induction of IgA secretion from IgA^+^ B cells caused by *L. plantarum* AYA.

### Gene Expression and Cytokine Production of DCs Separated from PP Cells in the Presence or Absence of L. plantarum AYA

**Figure 4:**
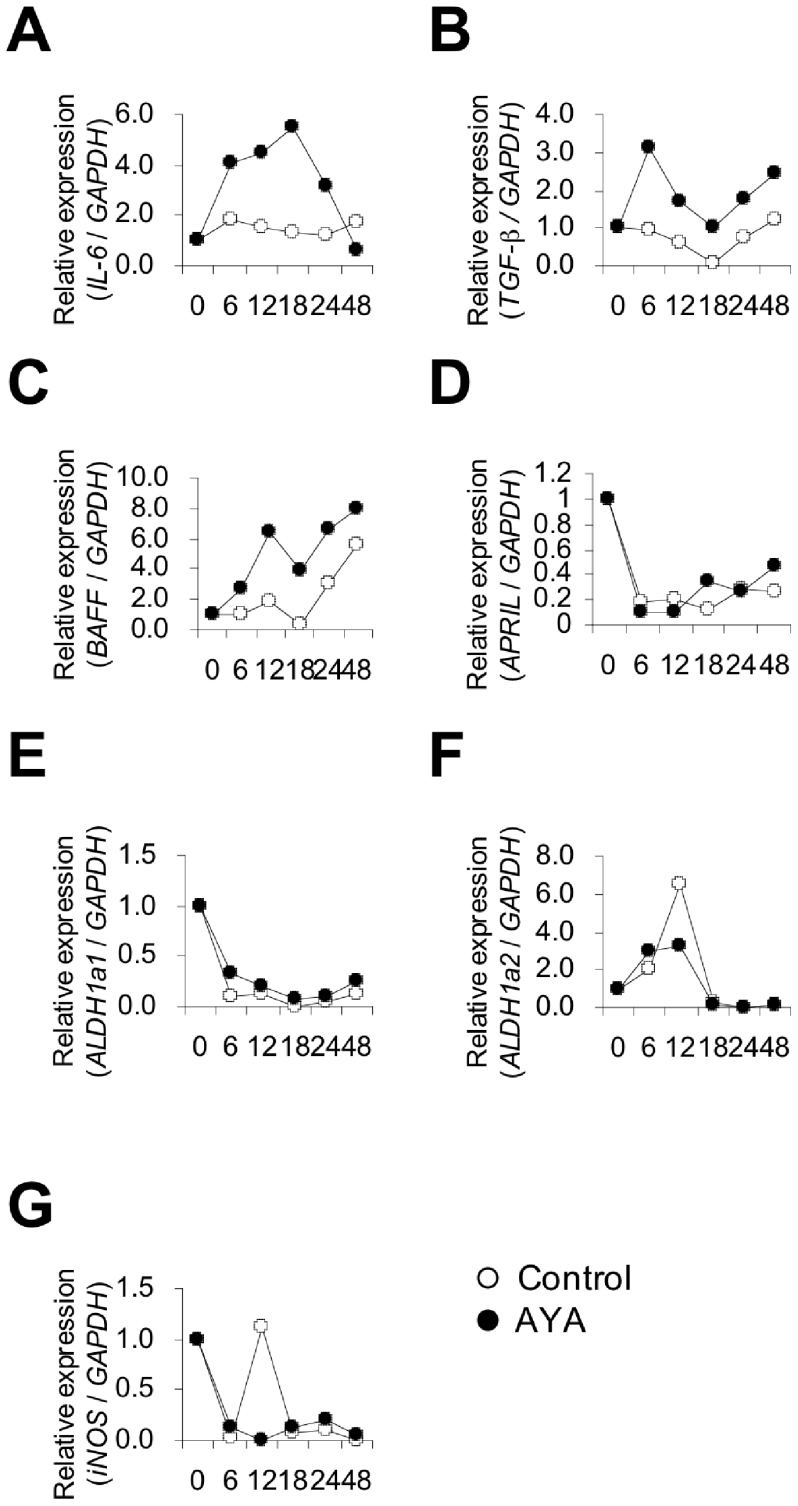
Expression of 7 genes was measured by real-time PCR. DCs from PP cells were cultured in the absence or presence of *L. plantarum* AYA for 6, 12, 18, 24, and 48 h. The level of gene expression was normalized to that of GAPDH mRNA expression in DCs from PP cells. A: IL-6, B: TGF-β, C:, BAFF, D: APRIL, E: ALDH1a1, F: ALDH1a2 and G: iNOS. Data represent one of two similar experiments.

DCs were separated from PP cells and cultured with or without *L. plantarum* AYA. We investigated the expression of TGF-β, iNOS, BAFF, and APRIL, retinoic acid synthase (ALDH1a1 and ALDH1a2), and IL-6 genes in DCs. As shown in Fig. 4A–G, expression of IL-6, TGF-β, and BAFF genes increased when DCs were incubated with *L. plantarum* AYA.

**Figure 5:**
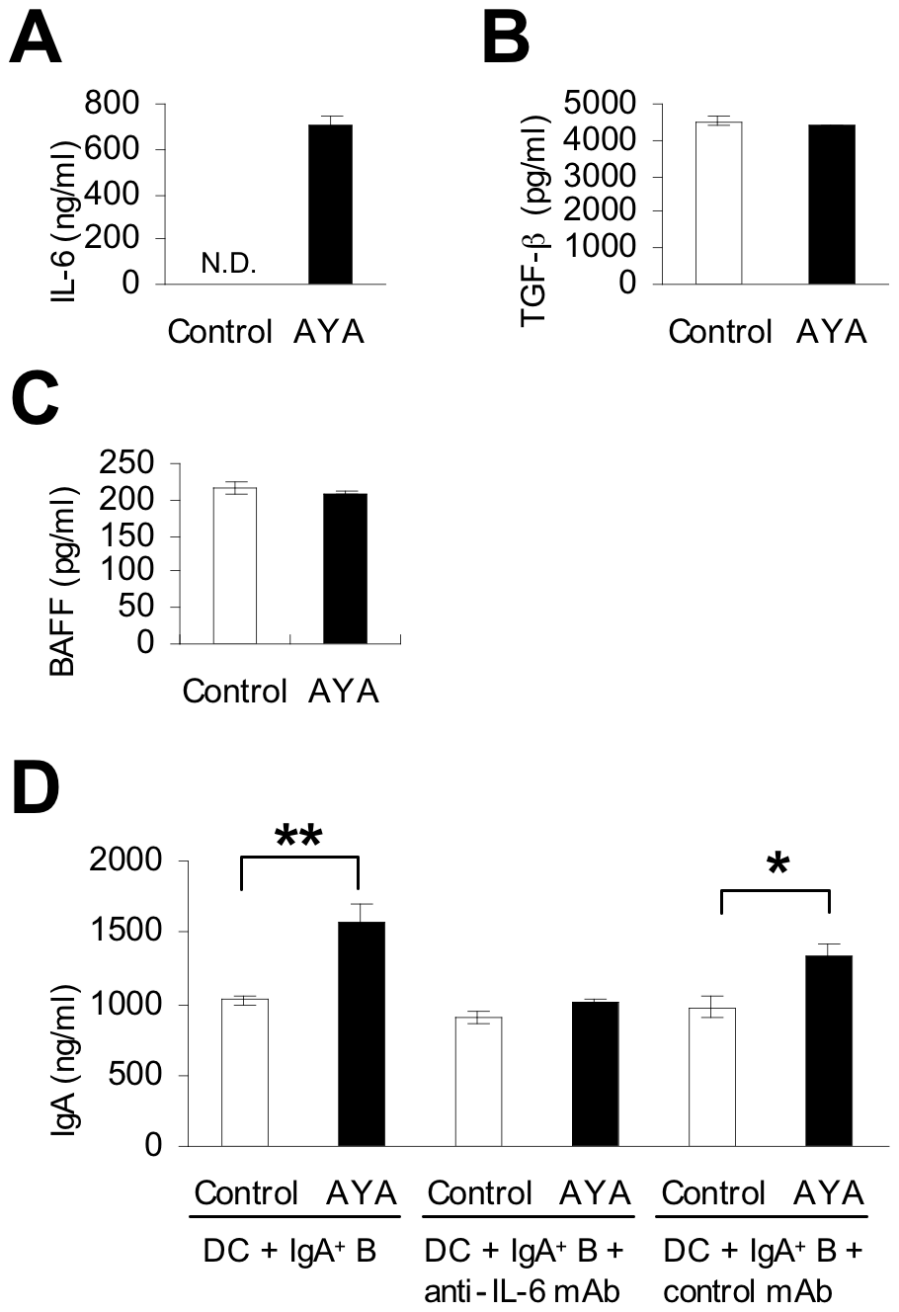
Cytokine and IgA production induced from PP cells with or without *L. plantarum* AYA. (A–C) DCs from PP cells were cultured in the absence or presence of *L. plantarum* AYA for 3 days. (A) IL-6 concentration was measured. Data are shown as mean ± SE. n = 3. (B) TGF-β concentration was measured. Data are shown as mean ± SE. n = 3. There were no statistically significant differences as determined by unpaired Student’s t test. (C) BAFF concentration was measured. Data are shown as mean ± SE. n = 3. There were no statistically significant differences as determined by unpaired Student’s t test. Data represent one of two similar experiments. (D) DCs and IgA^+^ B cells from PP cells were incubated with anti-IL-6 mAb or a control mAb with or without *L. plantarum* AYA. IgA concentrations from culture supernatants were measured by ELISA. Data are shown as mean ± SE. n = 3. One asterisk indicate unpaired Student’s t test; P value is <0.05, Two asterisks indicate unpaired Student’s t test; P value is <0.01. Data represent one of two similar experiments.

We measured IL-6, TGF-β, and BAFF production after 4-day incubation of DCs in the presence or absence of *L. plantarum* AYA. We found that IL-6 concentration in DCs cultured in the presence of *L. plantarum* AYA was significantly higher than that in DCs cultured without the bacterium (Fig. 5A). However, TGF-β and BAFF concentrations in DCs were not statistically different in the presence or absence of *L. plantarum* AYA (Fig. 5B–C).

To confirm that endogenous *L. plantarum* AYA-induced IL-6 secretion by DCs was involved in IgA production, purified DCs were incubated for 7 days with IgA^+^ B cells separated from PP cells with or without a neutralizing anti–IL-6 mAb. IgA production induced by *L. plantarum* AYA in cultures of PP DCs and IgA^+^ B cells was completely inhibited by the addition of anti–IL-6 mAb but not by control mAb (Fig. 5D).

### Effect of Oral Administration of L. plantarum AYA on Virus Infection

**Figure 6:**
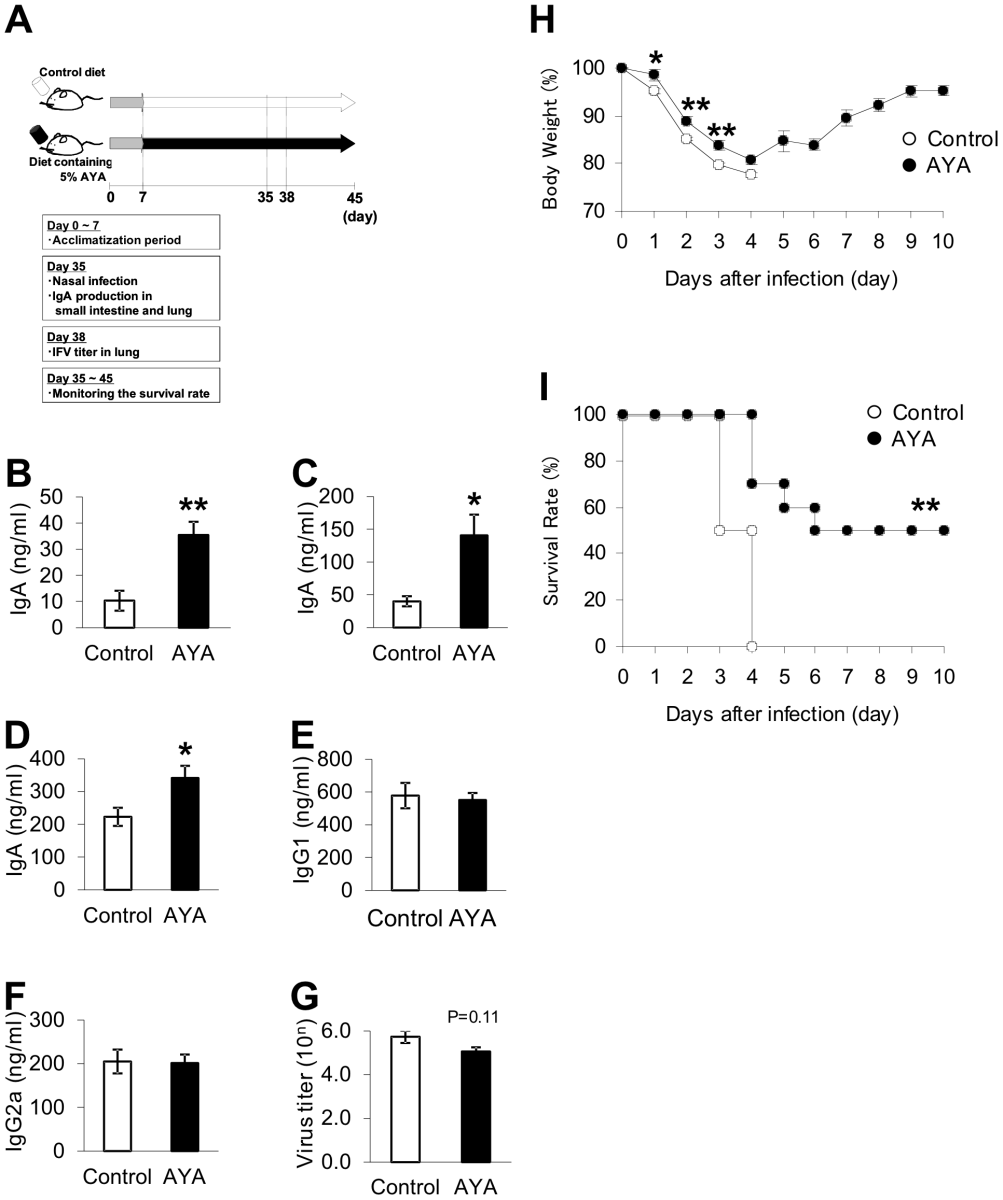
The effect of oral administration of *L. plantarum* AYA. Forty BALB/c mice were fed a common diet for 7 days. Then, 20 BALB/c mice per group were fed a control diet or a diet containing *L. plantarum* AYA for 28 days. After 35 days, 5 mice in each group were sacrificed, and the washings from the small intestine and BALF were collected. Fifteen mice in each group were subsequently infected with X-31 virus. After 38 days, 5 mice in each group were dissected, and BALF was collected. The remaining 10 mice per group were monitored for the survival rate for 10 days after infection. (A) The experimental procedure. (B) IgA concentration in the washings from the upper small intestine at 35 days. Data are shown as mean ± SE. n = 5. Two asterisks indicate unpaired Student’s t test; P value is <0.01. (C) IgA concentration in the washings from the lower small intestine at 35 days. Data are shown as mean ± SE. n = 5. Asterisk indicate unpaired Student’s t test; P value is <0.05. (D) IgA concentration in BALF at 35 days. Data are shown as mean ± SE. n = 5. Asterisk indicate unpaired Student’s t test; P value is <0.05. (E) IgG1 concentration in BALF at 35 days. Data are shown as mean ± SE. n = 5. (F) IgG2a concentration in BALF at 35 days. Data are shown as mean ± SE. n = 5. (G) Titer of X-31 virus in BALF of mice at 38 days. Data are shown as mean ± SE. n = 5. (H) Change of body weight. The humane endpoint is 20% body weight loss. Data are shown as mean ± SE. n = 5. One asterisk indicate unpaired Mann–Whitney’s U test; P value is <0.05, Two asterisks indicate unpaired Mann–Whitney’s U test; P value is <0.01. (I) Survival rate of mice in each group. Data are shown as mean ± SE. n = 10. Two asterisks indicate unpaired the Kaplan–Meier test; P value is <0.01. Data represent one of two similar experiments.

We investigated the effect of oral administration of *L. plantarum* AYA using the experimental procedure shown in Fig. 6A. Each group contained 20 mice aged 7 weeks, fed either a normal diet (AIN93G) or diet containing 5% *L. plantarum* AYA for 4 weeks. Five mice in each group were then sacrificed to study IgA production in the small intestine and lung. IgA concentrations in the small intestine and BALF were measured in the two groups. IgA concentration in the small intestine of the *L. plantarum* AYA diet group was 3.5-fold higher than that of the normal diet group. This difference was statistically significant (Fig. 6B–C: Washings from the upper small intestine, P<0.01; washings from the lower small intestine, P<0.05). In BALF, total IgA concentration in the *L. plantarum* AYA diet group was 1.5-fold higher than that in the control diet group, and this difference was statistically significant (P<0.05) (Fig. 6D), but the X-31 virus specific IgA levels of the *L. plantarum* AYA diet group and the control diet group were all below the detection limit. There was no difference of IgG1 and IgG2a concentration between both groups (Fig. 6 E–F).

We inoculated the remaining mice with 5 LD_50_ of influenza X-31 virus. The lung is the target organ of IFV. We then examined the virus titer in BALF on the third day after inoculation. The average virus titer in the control and *L. plantarum* AYA diet groups was 10^6.0^ and 10^5.3^, respectively (Fig. 6G). Although the difference was not significant (P = 0.11), the virus titer in the *L. plantarum* AYA diet group tended to be lower than that in the control diet group.

We then investigated whether oral administration of *L. plantarum* AYA affected the survival rate of mice inoculated with X-31. We monitored body weight daily for 10 days and set up the humane endpoint as 20% body weight loss after IFV infection (Fig. 6H). The body weight of the mice fed *L. plantarum* AYA diet was significantly higher than that of the normal diet group at days 1–3. A 50% survival rate was observed 10 days after the X-31 virus challenge in mice fed the *L. plantarum* AYA diet. In contrast, none of the mice fed the normal diet survived for more than 10 days after the challenge. The survival rate of mice in the *L. plantarum* AYA diet group was therefore significantly higher than that in the normal diet group (P<0.01) (Fig. 6I).

## Discussion

LAB such as certain *Lactobacillus* and *Bifidobacterium* species have been reported to modulate mucosal and systemic immune responses [17]–[19]. It is known that LAB enhance mucosal IgA production. *Lactobacillus* GG is associated with increased IgA production [20], whereas *L. johnsonii* (NCC 533) causes increased IgA production in PP whole-organ culture supernatants [21]. The mechanism by which LAB induce IgA production and the genera of LAB that effectively induce this production remains unclear. The present study showed that *L. plantarum* AYA, selected from 140 LAB strains, induced IgA production and protected against IFV infection.

In previous studies, the cells associated with IgA induction in PPs following oral administration of LAB remained unknown. Mucosal plasma cells originate mainly from homing IgA-committed B cells, which undergo IgM-to-IgA isotype class switching at inductive sites of mucosal immunity, such as PP and NALT [10]–[12]. This process of B-cell differentiation, including class switching, is essential for inducing IgA expression at the mucosal surface. We have demonstrated previously that PP CD11b^+^ DC induces naive B cells to produce higher concentrations of IgA than SP CD11b^+^ DC [15]. Natural secretory IgA is induced by constant antigenic stimulation by intestinal commensal bacteria, with DCs playing an important role in this process [22], [23]. From these reports, we hypothesized that DCs may be involved in the mechanism that results in increased IgA production by *L. plantarum* AYA. To confirm this hypothesis, we performed the following experiments.

Figure 2A–D shows that the proportion of IgA^+^B220^+^ cells was higher in mice fed *L. plantarum* AYA than in mice not inoculated with the organism. However, the total IgA concentration in supernatants of DCs and IgM^+^B cells cultured with *L. plantarum* AYA was not statistically different from that in supernatants of these cells grown in the absence of *L. plantarum* AYA (data not shown). These data suggest that *L. plantarum* AYA induces an IgM-to-IgA class switch recombination *in vivo*, although the mechanism of this switch remains unclear.

On the other hand, we found that *L. plantarum* AYA enhanced IgA secretion from IgA^+^ B cells in the presence of DCs. This finding suggested that AYA had a direct impact on DCs separated from PP cells and that DCs had a direct impact on IgA^+^ B cells to promote IgA production (Fig. 3). Multiple factors enhancing IgA production have been reported. Of these, IL-6 enhances intestinal IgA production by promoting the differentiation of IgA^+^ B cells into plasma cells 15,23–25. We have previously demonstrated that PP CD11b^+^ DCs enhance IgA production through IL-6 secretion [15]. To determine the mechanism by which DCs induce the differentiation of IgA^+^ B cells into plasma cells, we focused on IL-6 production in DCs separated from PP cells. We observed that production of IL-6 from DC in the presence of *L. plantarum* AYA was significantly higher than that in its absence (Figs. 4A, 5A). Induction of IgA production by *L. plantarum* AYA was completely prevented by the addition of anti–IL-6 mAb (Fig. 5D). Our results suggest that *L. plantarum* AYA induces IL-6 production in DCs separated from PP cells and that IL-6 production promotes differentiation of IgA^+^B cells into plasma cells that induce IgA production.

Other factors that enhance IgA production have been reported. TGF-β induces IgA-specific T-cell–dependent class switch recombination [22], [26], [27]. APRIL and BAFF also act to induce IgM to IgA class switch recombination [28]–[30], whereas retinoic acid confers gut-homing properties to IgA^+^ B cells [14], [31]. In addition, Tezuka et al. [16] demonstrated that iNOS regulates IgA class switch recombination through expression of the TGF-β receptor and production of APRIL and BAFF. In our study, we observed that gene expression and production of APRIL, ALDH1a1, ALDH1a2, and iNOS were not increased when DCs were incubated with *L. plantarum* AYA (Figs. 4, 5). We also demonstrated that production of TGF-β and BAFF was not increased (Fig. 5) although gene expression was increased (Fig. 4). We intend to carry out further investigations on the production of TGF-β and BAFF.

IFV infection is a significant cause of morbidity and mortality worldwide, because of which preventive treatments are needed. It has been reported that oral and intranasal administration of LAB protects against IFV infection [8], [9], although the mechanism of this protection remains unclear.

We observed a statistically significant increase in total IgA production in the small intestine and lungs of mice fed *L. plantarum* AYA (Fig. 6B–D). There was no difference of IgG1 and IgG2a concentration between both groups (Fig. 6 E–F). We investigated whether *L. plantarum* AYA activated splenic natural killer cells in vitro, but there was no effect (data not shown). The virus titer in the lung in the *L. plantarum* AYA diet group tended to be lower than that in the normal diet group (Fig. 6G). We also observed that oral administration of *L. plantarum* AYA protected against influenza infection (Fig. 6H–I). We therefore consider that the possible protective mechanism against IFV infection resulting from oral administration of *L. plantarum* AYA may involve induction of IgA production in the lung. We could not compare the X-31 virus specific IgA production levels between the two groups, because they were all below detection limit. BALF is diluted considerably upon preparation, which may have resulted in difficulty to detect binding to the virus. Although not detected, we consider the possibility that the enhancement of IgA production of various specificities may have resulted in greater binding of IgA to IFV, resulting in the reduction of viral infection, contributing to the improved survival rate.

Ishida et al. reported that the immunostimulatory activities of LAB are dependent on the strain rather than the species [32]. In this study, 45 *L. plantarum* strains were tested, and a wide range of IgA induction values (82–1923 ng/ml) was observed. Our results appear to support the findings reported by Ishida et al.

Strain 63 induced the highest levels of IgA production in PP cells in vitro (Fig. 1A), but *L. plantarum* AYA induced the highest levels of IgA production in vivo (Fig. 2). Several possible reasons for this can be considered. One may be that strain 63 is more strongly affected by digestive enzymes. Another possibility may be that the transcytosis of strain 63 across the gut epithelium by M cells is inefficient.

In the present study, we used heat-killed preparations of LAB. If the mice were administrated live *L. plantarum* AYA, they may possibly proliferate in the intestinal tract. If this should happen, the dose required for live *L plantarum* AYA, may be less than that for killed *L plantarum* AYA.

In conclusion, we selected *L. plantarum* AYA from 140 LAB strains, and clarified the mechanism by which *L. plantarum* AYA-induced IgA production through stimulation of DC. We also showed that oral administration of *L. plantarum* AYA enhanced immune responses in the intestine and lung and protected against IFV infections.
